# Effects of Ramadan intermittent fasting on North African children’s heart rate and oxy-haemoglobin saturation at rest and during sub-maximal exercise

**DOI:** 10.5830/CVJA-2016-078

**Published:** 2017

**Authors:** Mohamed Amine Fenneni, Imed Latiri, Sonia Rouatbi, Helmi Ben Saad, Mohamed Amine Fenneni, Asma Aloui, Sonia Rouatbi, Helmi Ben Saad, Karim Chamari, Helmi Ben Saad

**Affiliations:** Laboratory of Physiology, Faculty of Medicine, University of Sousse, Tunisia; Laboratory of Physiology, Faculty of Medicine, University of Sousse, Tunisia; Laboratory of Physiology, Faculty of Medicine, University of Sousse, Tunisia; Laboratory of Physiology, Faculty of Medicine, University of Sousse, Tunisia; Faculty of Sciences, Bizerte, Carthage University, Tunisia; High Institute of Sport and Physical Education, University of Gafsa, Gafsa, Tunisia; Department of Physiology and Functional Exploration, Farhat Hached University Hospital of Sousse, Tunisia; Department of Physiology and Functional Exploration, Farhat Hached University Hospital of Sousse, Tunisia; Athlete Health and Performance Research Center, ASP ETAR , Qatar Orthopedic and Sports Medicine Hospital, Qatar; Research Laboratory LR14ES05, Faculty of Medicine, University of Sousse, Tunisia

**Keywords:** Tunisia, Ramadan fasting, heart rate, oxyhaemoglobin saturation, walking test, child

## Abstract

**Aim:**

To examine the effects of Ramadan intermittent fasting (RIF) on the heart rate (HR) and oxyhaemoglobin saturation levels (oxy-sat) of boys at rest and during a six-minute walking test (6MWT).

**Methods:**

Eighteen boys (age: 11.9 ± 0.8 years, height: 153.00 ± 8.93 cm, body mass: 55.4 ± 18.2 kg), who fasted the entire month of Ramadan in 2012 for the first time in their lives, were included. The experimental protocol comprised four testing phases: two weeks before Ramadan (pre-R), the end of the second week of Ramadan (R-2), the end of the fourth week of Ramadan (R-4), and 10 to 12 days after the end of Ramadan (post-R). During each phase, participants performed the 6MWT at approximately 15:00. HR (expressed as percentage of maximal predicted HR) and oxy-sat (%) were determined at rest and in each minute of the 6MWT.

**Results:**

R-4 HR values were lower than those of (1) pre-R (in the second minute), (2) R-2 (in the first and second minutes), and (3) post-R (in the first, second, fourth, fifth and sixth minutes). R-2 oxy-sat values were higher than those of pre-R (in the third minute) and those of post-R (in the fifth minute). Post-R oxy-sat values were lower than those of pre-R and R-4 in the fifth minute. These oxy-sat changes were not clinically significant since the difference was less than five points.

**Conclusion:**

In non-athletic children, their first RIF influenced their heart rate data but had a minimal effect on oxy-sat values.

## Aim

Ramadan intermittent fasting (RIF) is the fourth pillar of Islam and, according to most religious scholars, it concerns every healthy Muslim after puberty.^1^ It is important to know the effects of RIF on human physiology, not only for Muslim-majority countries but also for many countries where Muslims are in the minority.^2^ In a globalised society, physicians have to deal with issues such as Muslim patients who desire to fast during Ramadan, regardless of whether they are in good health or not.^3^

Several studies have assessed the effects of RIF on variables such as body mass, biological data or markers, and sleep patterns in healthy adult subjects.^2^,^4^-^10^ However, only a few studies have been performed on healthy children.^11^-^14^ This lack of information on the effects of RIF on the health and physical performance of healthy children could lead to confusion worldwide. For example, on 10 June, one week before the start of Ramadan in 2015, the Barclay Primary School in east London, UK, sent a letter to the parents of its students (http://www.telegraph.co.uk/news/religion/11669767/Primary-schools-ban-children-fromfasting-during-Ramadan.html; visited 8 May 2016): ‘The policy of both Barclay Primary School and all schools within the Lion Academy Trust does not allow any children (aged 6 to 12 years) attending the schools to fast’, suggesting that fasting would endanger the health and education of students.

In children, the evaluation of heart rate (HR) and/or oxyhaemoglobin saturation (oxy-sat) dynamics during exercise provides valuable information for making management decisions, resulting in improved quality of life and functional capacity.^15^,^16^ During exercise, HR responses reflect subjects’ physical level and aerobic fitness.^17^,^18^ To the best of our knowledge, to date, all studies interested in the effects of RIF on HR changes concern adults^19^-^25^ and none has been performed in children.^26^ In addition, conclusions concerning the effect of fasting on healthy adults’ HR changes during exercise were controversial,^19^-^25^ with modified (lower^19^,^24^ or higher^25^) or unchanged values reported.^20^, ^23^

Oxygen desaturation provides information regarding exerciseinduced desaturation.^17^ However studies on the RIF effects on oxyhaemoglobin saturation seem non-existent. A Medline search (performed on 28 January 2015) using as keywords ‘fasting’ and ‘oximetry or oxyhemoglobin saturation’ and ‘exercise’ found no articles.

In the four studies concerning the effects of RIF on the sports performance of healthy children, no information was given on HR and/or oxy-sat.^11^-^14^ In that regard, a recent article^26^ has described and criticised these publications,^11^-^14^ and the authors recommend studies focusing on the effects of RIF on HR and oxy-sat.^26^ Therefore, the aim of our study was to examine the effects of RIF on HR and oxy-sat, determined at rest and during a field exercise test in untrained Tunisian boys.

## Methods

Part of this study’s methodology was previously described in an article reporting on the effects of RIF on first-time fasting boys’ performance in short-term explosive exercises, as well as in sub-maximal endurance exercise.^12^ The major details concerning the applied methodology are presented below.

Eighteen healthy non-athletic boys who fasted for the first time in their lives during Ramadan 2012 were included. The protocol was approved by the local hospital ethics committee, and written informed consent was obtained from all children and their parents. The boys were asked to avoid strenuous activities 24 hours before each testing phase. Participants were informed that their participation was free of pressure and that they could withdraw from the study at any time (including that they could decide to stop fasting).

The experimental design consisted of four testing phases: two weeks before Ramadan (pre-R), the end of the second week of Ramadan (R-2), the end of the fourth week of Ramadan (R-4), and 10 to 12 days after the end of Ramadan (post-R). The decimal age (accuracy of 0.10 years) was calculated from the date of measurement and the date of birth. Body mass (± 1 kg) was measured during each phase and height was measured to the nearest 0.1 cm.^12^

The boys were familiarised with the six-minute walking test (6MWT) to minimise the learning effect.^27^ 6MWTs were conducted at the same time of day in the interval between 15:00 and 17:00.^28^ This period corresponded to the last fasting hours of the day, with the fast break time ranging from 19:31 at the beginning to 19:04 at the end of Ramadan 2012 at the location of the study. During each testing phase, each boy performed one 6MWT.

The following data were collected/calculated: six-minute walking distance (6MWD, in m, % of predicted value^27^), oxy-sat (%) and HR [bpm, % of maximal predicted HR (= 208–0.7 × age)^29^] determined at rest and in each minute of the 6MWT, and the 6MWD × sixth minute oxy-sat index (m).^17^,^18^ The 6MWTs were performed according to international guidelines.^30^ At the end of the 6MWT, the 6MWD (m) was noted. HR (Polar RS 800, Polar Electro Oy, Kempele, Finland) and oxy-sat (Nonin Medical, Inc, Minneapolis, MN) were recorded at rest and in each minute of the 6MWT. Additional 6MWT methodology details have been presented in a separate publication.^12^

## Statistical analysis

Data are presented as mean values ± standard deviation (SD) for anthropometric data and mean ± SD (95% confidence interval) for HR, oxy-sat and the 6MWD × sixth minute oxy-sat index. The Kolmogorov–Smirnov test for normality revealed that the data were normally distributed. Analysis of variance (ANOVA) was then conducted to compare the HR and oxy-sat data measured at the seven time points of the 6MWT (rest, first, second, third, fourth, fifth and sixth minutes) during the four testing phases. ANOVA was also conducted to compare the 6MWD × sixth minute oxy-sat index in the four phases. When appropriate, significant differences between means were tested using the Tukey post hoc test. Statistical analyses were performed using Statistica software (Statistica Kernel version 6; StatSoft, Paris, France). Significance was set at p < 0.05.

## Results

The mean ± SD of the 18 boys’ age, height and body mass were 11.9 ± 0.8 years, 153.00 ± 8.93 cm and 55.4 ± 18.2 kg, respectively.

**Fig. 1.**
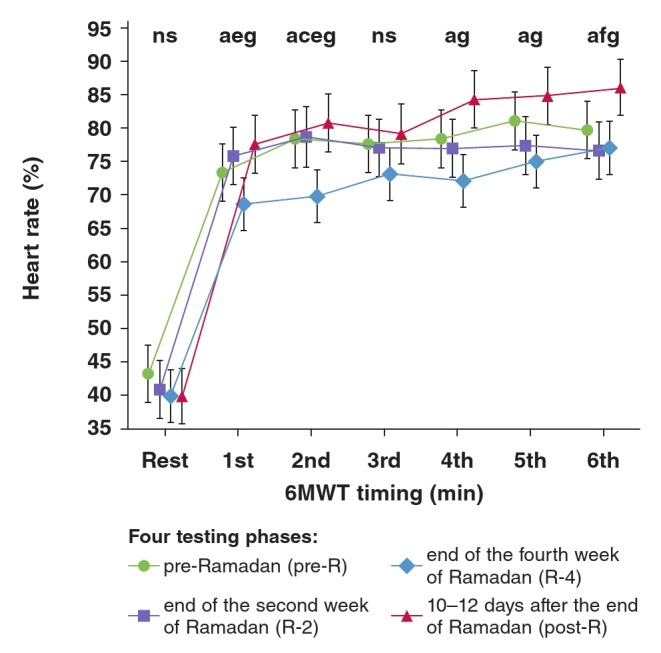
Effect of Ramadan intermittent fasting on heart rate determined at rest and in each minute of the six-minute walking test (6MWT) in 18 non-athletic boys fasting for the first time. Heart rate data is expressed as percentage of maximal predicted heart rate. Mean values are shown. Error bars represent 95% confidence intervals. ns: non-significant. ^a^p < 0.05: ANOVA between the four testing phases for the same timing; bp < 0.05 (Tukey post hoc test): pre-R vs R-2 for the same timing; cp < 0.05 (Tukey post hoc test): pre-R vs R-4 for the same timing; ^dp^ < 0.05 (Tukey post hoc test): pre-R vs post-R for the same timing; ep < 0.05 (Tukey post hoc test): R-2 vs R-4 for the same timing; fp < 0.05 (Tukey post hoc test): R-2 vs post-R for the same timing; gp < 0.05 (Tukey post hoc test): R-4 vs post-R for the same timing.

The effect of RIF on HR: Fig. 1 shows the HR data (%) determined during the four testing phases at the seven time points of the 6MWT. There was no significant difference between the four testing phases in resting or third-minute HR values [ANOVA (n =18, df = 3, F = 0.82) and ANOVA (n = 18, df = 3, F = 1.253), respectively]. However, there were significant differences between the four testing phases in the:

• first-minute 6MWT HR values [ANOVA (n = 18, df = 3, F = 4.32, p < 0.008)]. HR was lower during R-4 (69 ± 7 bpm) compared to R-2 (76 ± 10 bpm; p = 0.04) and post-R (78 ± 6; p = 0.008).
• second-minute 6MWT HR values [ANOVA (n = 18, df = 3, F = 5.447, p < 0.0022)]. HR was lower during R-4 (70 ± 9 bpm) compared to pre-R (78 ± 9 bpm; p = 0.029), R-2 (79 ± 10 bpm; p = 0.027) and post-R (81 ± 7 bpm; p = 0.003).
• fourth-minute 6MWT HR values [ANOVA (n = 18, df = 3, F = 4.83, p < 0.0045)]. HR was lower during R-4 (72 ± 9 bpm) compared to post-R (84 ± 6 bpm; p = 0.002).
• fifth-minute 6MWT HR values [ANOVA (n = 18, df = 3, F = 4.054, p < 0.01)]. HR was lower during R-4 (75 ± 7 bpm) compared to post-R (85 ± 7 bpm; p = 0.009).
• sixth-minute 6MWT HR values [ANOVA (n = 18, df = 3, F = 3.43, p < 0.023)]. HR was higher during post-R (86 ± 9 bpm) compared to R-2 (77 ± 10 bpm; p = 0.04) and R-4 (77 ± 8 bpm; p = 0.04).

**Fig. 2.**
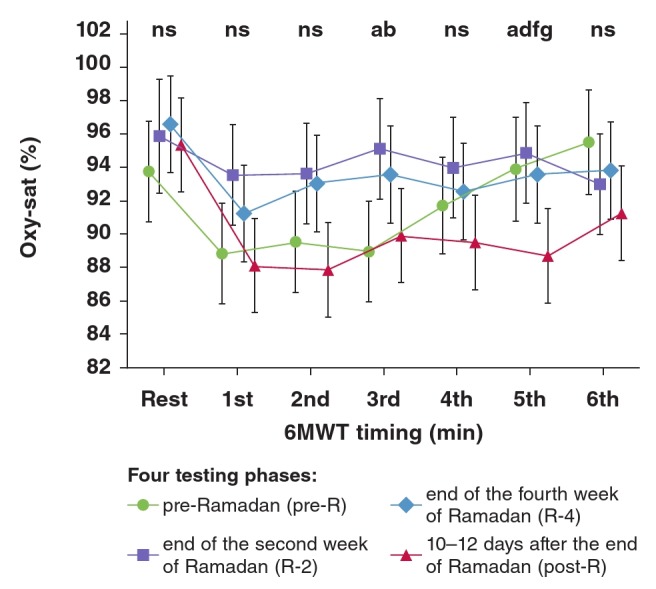
Effect of Ramadan intermittent fasting on oxyhaemoglobin saturation (oxy-sat; %) determined during the six-minute walking test (6MWT) in 18 non-athletic boys fasting for the first time. Mean values are shown. Error bars represent 95% confidence intervals. ns: non-significant. ^a^p < 0.05: ANOVA between the four testing phases for the same timing; bp < 0.05 (Tukey post hoc test): pre-R vs R-2 for the same timing; cp < 0.05 (Tukey post hoc test): pre-R vs R-4 for the same timing; dp < 0.05 (Tukey post hoc test): pre-R vs post-R for the same timing; ^e^p < 0.05 (Tukey post hoc test): R-2 vs R-4 for the same timing; ^f^p < 0.05 (Tukey post hoc test): R-2 vs post-R for the same timing; gp < 0.05 (Tukey post hoc test): R-4 vs post-R for the same timing.

The effect of RIF on oxy-sat: Fig. 2 shows the oxy-sat data (%) obtained during the four phases at the seven time points of the 6MWT. There was no significant difference between the four testing phases in resting or first, second, fourth and sixth minutes oxy-sat values [ANOVA: (n = 18, df = 3, F = 3.52), (n = 18, df = 3, F = 1.83), (n = 18, df = 3, F = 2.12), (n = 18, df = 3, F = 1.41) and (n = 18, df = 3, F = 2.13), respectively]. However, there were significant differences between the four testing phases in the:

• third-minute oxy-sat values [ANOVA (n = 18, df = 3, F = 4.07, p < 0.01)]. The Tukey test showed a significant difference between pre-R and R-2 [89 ± 7 vs 95 ± 2%; p = 0.02].
• fifth-minute oxy-sat values [ANOVA (n = 18, df = 3, F = 4.55, p < 0.006)]. The Tukey test showed a significant difference between pre-R and post-R (94 ± 2 vs 89 ± 7%; p = 0.04), between R-2 and post-R (95 ± 3 vs. 89 ± 7%; p = 0.008), and between R-4 and post-R (94 ± 6 vs 89 ± 7%; p = 0.04).

**Fig. 3.**
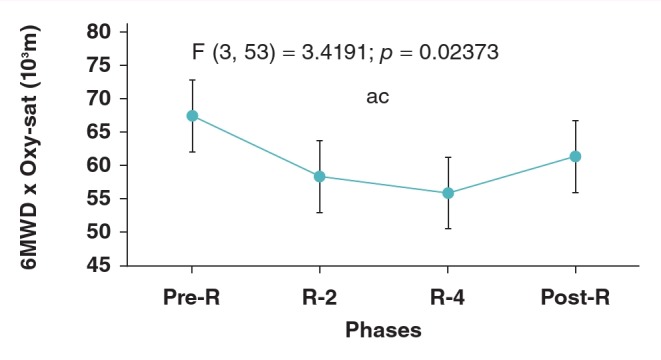
Effect of Ramadan intermittent fasting on the product of oxyhaemoglobin saturation (oxy-sat) and the sixminute walking distance (6MWD) determined during the sixth minute of the six-minute walking test (6MWT) in 18 non-athletic boys fasting for the first time. Mean values are shown. Error bars represent 95% confidence-intervals. ^a^p < 0.05: ANOVA between the four testing phases; ^b^p < 0.05 (Tukey post hoc test): pre-R vs R-2; ^c^p < 0.05 (Tukey post hoc test): pre-R vs R-4; ^d^p < 0.05 (Tukey post hoc test): pre-R vs post-R; ^e^p < 0.05 (Tukey post hoc test): R-2 vs R-4; ^f^p < 0.05 (Tukey post hoc test): R-2 vs post-R; ^g^p < 0.05 (Tukey post hoc test): R-4 vs post-R.

Fig. 3 shows the 6MWD × oxy-sat indices calculated in the sixth minute of each 6MWT. A significant difference between the four testing phases was found [F (3, 53) = 3.4191; p = 0.023]. The Tukey test showed a significant difference (p = 0.019) between pre-R (67 573 ± 7 514 m) and R-4 (56 224 ± 12 274 m) values 

## Discussion

The objective of this study was to evaluate the effects of RIF on HR and oxy-sat data, determined at rest and during a sub-maximal field test in healthy, untrained boys fasting for the first time in their lives during Ramadan 2012. With regard to HR, R-4 values were lower than those of pre-R (second minute), R-2 (first and second minutes) and post-R (first, second, fourth, fifth and sixth minutes), with no significant difference between the four testing phases in resting and third-minute HR values. Concerning oxy-sat, R-2 values were higher than those of pre-R (third minute) and post-R (fifth minute), and post-R values were lower than those of pre-R and R-4 (fifth minute).

To the best of our knowledge, only four studies11-14 have recently described the effects of RIF on the exercise performance of healthy children. Our study included boys who lived in Kalaa- Kebira (Sousse region), a small town on the Tunisian east coast, known to have a low level of pollution. Fenneni et al.^26^ criticised these four articles and recommended studies focusing on the effect of RIF on physiological parameters such as HR and oxy-sat. They discussed in detail the required sample size, study design, 6MWT choice and procedures.^26^

RIF is not obligatory for pre-pubescent children, and many observe it from early adolescence.^12^,^26^ However, even though Islam requires such a practice only after puberty, it is a relatively frequent practice that pre-pubescent children attempt to fast the whole of Ramadan.^12^,^26^

## Effect of RIF on HR

RIF seemed to have a significant effect on HR, determined during the 6MWT. Our study showed that R-4 HR values were lower than those of pre-R (second minute), R-2 (first and second minutes), and post-R (first, second, fourth, fifth and sixth minutes), with no significant difference between the four testing phases in resting and third-minute HR values (Fig. 1). To our knowledge, this study is the first to examine the impact of RIF on the HR of healthy children.

Our results are in line with those observed in some studies conducted on adults,^19^,^24^ but opposite to those of Zerguini et al.^25^ Husain et al.^19^ showed that resting HR in adults was markedly lowered in sedentary fasting male subjects (aged 20–45 years) during the month of Ramadan, while responses in females were only slightly decreased. Ramadan and Barac-Nieto^24^ found a small but significant HR reduction in response to sub-maximal exercise during the month of Ramadan in sedentary males aged 35 ± 2 years. Zerguini et al.^25^ found that HR measured after a 12-minute run was higher during R-4 than pre-R in professional soccer players aged 17–34 years. Karli et al.^22^ observed that peak HR values determined after a 30-second Wingate test were not significantly different between Ramadan and pre-R in elite power athletes aged 20–40 years. Brisswalter et al.^23^ found that maximal HR recorded after an incremental maximal running test was not modified during R-4 (vs pre-R) in well-trained runners aged 24 ± 3 years. Also, Güvenç^21^ found that HR at eight, 10, 11 and 12 km/h of a modified 20-m shuttle-run test and peak HR after this exercise were unchanged during Ramadan in male soccer players aged 17 ± 1 years. Finally, HR during a 60-minute endurance treadmill running test,^31^ and a multi-stage fitness test20 remained unaffected during Ramadan in moderately trained active men (mean age: 27 ± 7 years) and in elite judokas (mean age: 18 ± 1 years), respectively.

There are three possible explanations as to how the decrease in HR during RIF could be explained:

• significant reduction in endurance capacity during Ramadan.^12^ In a previous study, it was shown that the 6MWD was lower during R-4 compared to pre-R but returned to baseline values post-R. Therefore, the RIF-induced attenuation of HR during walking tests should be taken into consideration when assessing human exercise capacity, applying the HR output relationship.
• changes in lifestyle during Ramadan.^12^ This mainly impacts on the schedule of food intake, which may affect HR^32^ Indeed, fasting lowers the metabolic rate due to the absence of digestion, which increases HR for two or three hours during the day.^33^ The possible role of plasma leptin and ghrelin concentrations (i.e. satiety and hunger hormones, respectively, known to affect cardiovascular activity^34^,^35^) as an explanation of HR reduction was rejected by Zoladz et al.,^36^ who found no effect of the overnight fast on pre- and during-exercise plasma leptin and ghrelin concentrations. However, this may be due to the relatively short duration of fasting in their study compared to RIF.^37^, ^38^ This possible explanation should be considered in future studies.
• reduced sympathetic tone during fasting.^19^ Zoladz et al.^36^ demonstrated a significant decrease in HR by about 10 bpm after an overnight fast. Their most likely explanation was the observed significant increase in plasma norepinephrine concentration, leading to an increase in systemic vascular resistance, loading of arterial baroreceptors, and causing vagal stimulation.^39^,^40^

The validity of the above explanations, taken from studies done in adults, should be demonstrated in children.

## Effect of RIF on oxy-sat

RIF seemed to have a statistically significant effect on oxy-sat determined during the third and fifth minutes of the 6MWT. R-2 oxy-sat values were higher than those of pre-R (third minute) and post-R (fifth minute), and post-R values were lower than those of pre-R and R-4 (fifth minute). These significant changes between phases were approximately five points (Fig. 2), and could not be considered clinically significant since that requires a change of more than five points.17,18,27 Therefore RIF slightly improved blood oxygenation and oxygen transport by haemoglobin, suggesting that the haemoglobin affinity with oxygen may have increased.^41^,^42^

Recently, a novel desaturation index (6MWD × oxy-sat) has been proposed, with the objective of improving the information obtained from the 6MWT.^17^,^18^ In our study, this index was lower during R-4 compared to pre-R (Fig. 3). However, during the four testing phases, the desaturation index was in the normal range (minimum–maximum: 32 305–78 720). In chronic respiratory dysfunction patients, a low 6MWD × oxy-sat product (e.g. 20 000 m) was related to a clearly increased hazard ratio for mortality,^43^ and it predicted quality of life.^44^

The rationale for HR and oxy-sat measurements in non-diseased children deals with children’s safety. For instance, during school sports practice late in the afternoon, especially when Ramadan occurs in summer, the length of the fast could potentially be a concern with young fasting children. Moreover, such information could help medical and educational authorities to make rational decisions concerning banning/allowing the practice of RIF on school grounds. It is of paramount importance to note that banning of such a religious practice could trigger sharp reactions from the concerned communities; therefore, any potential banning has to be based on sound and powerful data.

## Limitations

The main limitation of the present study, as observed in previous ones on RIF effects on the physical capacities of healthy children,^11^-^14^,^26^ was the absence of a non-fasting control group. The inclusion of such a group may decrease the risk of learning effects skewing the findings, and circumvent any hazard to the internal strength of the results.^12^,^26^ This crucial point was previously discussed.^12^,^26^ The second limitation was not measuring the walked distance in each minute of the 6MWT. Therefore, the correlation between HR and the 6MWD in each minute of the 6MWT was not studied. However, as recommended, the boys were invited to walk as far as possible in six minutes along a flat hallway.30 In addition, none of them needed to rest since they knew how to pace themselves, based on pre-experiment tests.^12^,^30^

## Conclusion

This study shows that summer RIF observed for the first time by non-athletic boys aged 12 years had a minimal effect on oxy-sat but significantly impaired HR during the 6MWT.
